# Effects of Koumiss on Intestinal Immune Modulation in Immunosuppressed Rats

**DOI:** 10.3389/fnut.2022.765499

**Published:** 2022-02-14

**Authors:** Qinyu Li, Chunjie Zhang, Tuya Xilin, Mingyue Ji, Xiangxi Meng, Yulian Zhao, Bateer Siqin, Na Zhang, Minhui Li

**Affiliations:** ^1^Department of Pharmacy, Baotou Medical College, Baotou, China; ^2^Center for Translational Medicine, Baotou Medical College, Baotou, China; ^3^Laboratory of Mongolian Medicine, Xilinguole Meng Mongolian General Hospital, Xilinhaote, China; ^4^Pharmaceutical Laboratory, Inner Mongolia Institute of Traditional Chinese Medicine, Hohhot, China; ^5^Inner Mongolia Key Laboratory of Characteristic Geoherbs Resources and Utilization, Baotou Medical College, Baotou, China; ^6^Office of Academic Research, Qiqihar Medical University, Qiqihar, China

**Keywords:** koumiss, intestinal immune, LC-MS/MS, cyclophosphamide, flow cytometry

## Abstract

Koumiss is a traditional fermented dairy product with health and medicinal benefits. It is very popular in the Inner Mongolia Autonomous Region of China. The results of relevant studies have shown that koumiss can regulate the gastrointestinal environment, improve the absorption of nutrients, improve the body's intolerance to lactose, enhance the body's immunity, prevent scurvy and atherosclerosis, and aid in the treatment of tuberculosis. However, there are no systematic reports on the effects of koumiss on immunity. In this study, we aimed to decipher the effects of koumiss on intestinal immune modulation. We used liquid chromatography-tandem mass spectrometry (LC-MS) analysis to determine the composition of Koumiss. Using Compound Discoverer software, we compared the mass spectrometry data with the compound information in the online databases ChemSpider and mzCloud to intelligently identify the main chemical components of koumiss. Additionally, we used Mass Frontier small molecule fragmentation library^TM^ to determine the structure of fragment ions. A total of 21 components were identified, which clarified the chemical basis of koumiss. These 21 compounds were then used to perform molecular docking with immune-related targets, such as TNF, IL2, IL10, etc. The results indicated good docking activity between most of the compounds and the targets. Then, an immunosuppressive rat model was used to determine the therapeutic effect of koumiss. The results of this study showed that koumiss could, to a certain extent, correct the atrophy of the thymus and spleen in immunosuppressed model rats. The number of leukocytes, lymphocytes, and the CD4^+^/CD8^+^ ratio of peripheral blood lymphocytes was also increased. In addition, it could effectively improve the structure of the small intestinal mucosa, which shows that koumiss has a positive effect on the intestinal immune function of immunosuppressed rats. These findings provide an experimental basis for the development and utilization of koumiss as a therapeutic product.

## Introduction

Koumiss is a dairy product made from fresh horse milk, and it contains a small amount of alcohol and is naturally fermented using the original mix of ferments (lactic acid bacteria and yeast) (1). Since the Yuan dynasty, the traditional Mongolian method of making koumiss and its use in treating a variety of diseases have been globally well-known (2). Koumiss is also a traditional drink of the nomadic people of Xinjiang and Inner Mongolia in northwest China. It is known to improve immunity and regulate homeostasis (3). Koumiss occupies a prominent place in Mongolian medicine as the first beverage used in food-based therapy (4). In addition to the Inner Mongolia International Mongolian Hospital and Xilinguole Institute of Mongolian Medicine, the Koumiss Medical Center has been established in Russia and Mongolia for assistance or treatment of chronic diseases, such as those of the digestive and cardiovascular system (5, 6).

**Figure 1:**
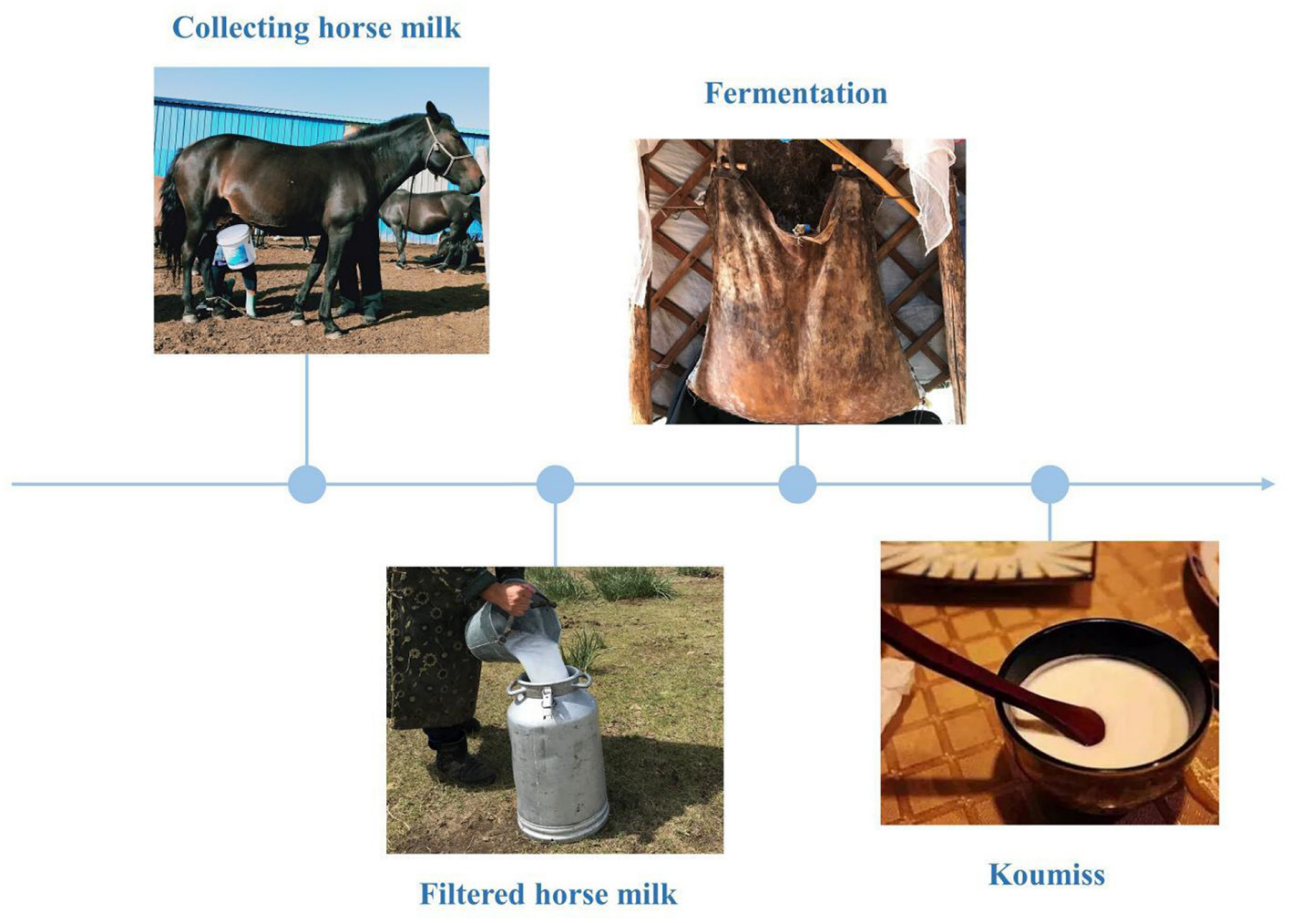
Traditional koumiss production process in the Xilinguole league of the Inner Mongolia Autonomous Region.

The existing body of research on koumiss suggests that can regulate the gastrointestinal environment, improve the body's absorption of nutrients, improve lactose intolerance (7), enhance immunity (8), prevent scurvy and atherosclerosis (9), and assist in the treatment of tuberculosis (10). These remedial properties of koumiss are attributed to its chemical composition and the traditional fermentation process. A survey of the traditional koumiss fermentation process in the Xilinguole region of Inner Mongolia shows that traditional koumiss is made by mixing fresh and filtered horse milk with 5% fermenting agent (i.e. koumiss) in a fermentation bucket, stirring it repeatedly with a wooden stick, and fermenting it at room temperature for 1-2 days (Figure 1). Additionally, koumiss has a complex micro-ecological environment, formed by the interaction of various species of organisms, including lactic acid bacteria and yeast, through a series of biochemical reactions, such as lactic acid and alcoholic fermentation. Koumiss has a higher nutritive value than fresh horse milk (11). The chemical composition of koumiss is the key to maintaining its quality. Koumiss is rich in free fatty acids, especially unsaturated fatty acids, containing approximately four to five times higher quantities than cow's milk; it also has a higher content of essential fatty acids, such as linoleic and linolenic acid, than that in cow's milk (12). Protein, lactose, minerals, vitamins, and amino acids are also present in high quantities in koumiss (12). Therefore, it is vital to understand the traditional processes and chemical composition of koumiss.

Traditional Mongolian medicine has developed the “koumiss therapy,” which has the therapeutic potential for immune system disorders; however, to date, there are no systematic studies on the effects of koumiss on human immunity. Therefore, in this study we aimed to identify the main components of koumiss (mainly from Xilinguole) *via* liquid chromatography-tandem mass spectrometry (LC-MS) in combination with Compound Discoverer software and Mass Frontier software, as well as its binding ability to immune-related proteins by molecular docking. An immunosuppressed rat model was used to investigate their therapeutic activities and evaluate parameters, including organ index, number of Peyer's nodes, and lymphocyte subpopulation. The effect of koumiss on the immune function of the body's intestinal tract was explored, providing a theoretical basis for the comprehensive development and utilization of koumiss.

## Materials and Methods

### Source of Koumiss

Koumiss was provided by Xilinguole Meng Mongolian General Hospital. Freeze-dry koumiss, and approximately 1 g of koumiss (equivalent to 20 mL of koumiss) lyophilized powder was weighed into a centrifuge tube, and 4 mL water was added to dissolve the powder. The solution was centrifuged at 3,000 rpm for 5 min. An equal volume of acetonitrile was added to the supernatant and the resulting solution was mixed using a vortex shaker for 30 s. After standing for 5 min, the mixture was shaken again for 30 s. The mixture was then centrifuged at 4 500 rpm for 10 min, the supernatant was collected, and an equal volume of acetonitrile was added to precipitate the protein; the above steps were then repeated. The final supernatant was passed through a 0.22-μm microporous filter membrane.

### LC-MS/MS Analysis of Koumiss

The samples were analyzed on a Thermo Scientific™ Ultimate™ 3000 RS system (Thermo Fisher Scientific, Waltham, MA, USA) coupled to a Q Exactive High-Resolution Benchtop Quadrupole Orbitrap mass spectrometer (Thermo Fisher Scientific) with a heated electrospray ionization source, and the system was controlled using the Xcalibur 2.3 software program (Thermo Fisher Scientific). Chromatographic separation was performed on a Hypersil GOLD C18 column (100 × 2.1 mm, 1.9 μm), with the column temperature maintained at 30°C. The mobile phase consisted of 0.1% formic acid in water **(A)** and acetonitrile **(B)** in the positive ion mode and the negative ion mode. The gradient conditions were as follows: 0–60 min, 5–95% B. The flow rate was maintained at 0.30 mL/min, and the injection volume was 2 μl. Full MS/dd-MS^2^ mode scan was used in positive ion mode and negative ion mode with a scan time of 60 min. The mass spectrometric settings for positive/negative ionization modes are listed in Table 1. All sample data were processed using Compound Discoverer software. The conditions were as follows: retention time range: 1–60 min; mass range: m/z 100–1000; mass tolerance: 5 ppm; area (max.) ≥1000000; and mzCloud Best Match ≥85. The data were processed for peak acquisition, and the m/z value, retention time, and signal intensity of each peak were listed to create a peak list by extracting the m/z and signal intensity values of the ion peaks. The data were compared with those in the online databases ChemSpider and mzCloud, which contain multi-level fragmentation mass spectra of compounds, for preliminary identification of each compound.

**Table 1:** Ion source, full MS, and dd-MS2 parameter of the Q-Exactive Orbitrap MS/MS.

**Ion source**
Sheath gas pressure	35 psi
Auxiliary gas flow rate	10 L/min
Spare gas flow rate	1 L/min
Spray voltage	+3,500 V
Capillary temperature	320°C
Probe heater temperature	350°C
S-Lens RF level	50 V
Full MS
Resolution	70,000
Mass range	150–1,500
AGC target	3e^6^
Maximum IT	100 ms
dd-MS^2^
Resolution	17,500
AGC target	1e^5^
Maximum IT	50 ms
Loop count	5
Mass isolation window	4.0 *m/z*
Normalized collision energy	30%
Minimum AGC	8e^3^
Intensity thresh	1.5e^5^
Exclude isotope	On
Dynamic exclusion	10 s

### Molecular Docking

Five immune-related targets (TNF, HLA-DRB1, FKBP1A, IL10, and IL2) were selected as receptors in the Genecards database, and 21 compounds were identified as ligands using Compound Discoverer software and Mass Frontier small molecule fragmentation library^TM^. The CAS numbers of drug small molecules were searched in the Pubchem database (https://pubchem.ncbi.nlm.nih.gov/), and 3D structures of drug small molecules were downloaded, energy minimized using Chem3D pro, and saved in mol2 format. Additionally, the 3D structure of the target protein was downloaded (PDB format) from PDB database (https://www.rcsb.org/). Then, the protein hydrolase was dehydrated using Pymol software, the original ligand of the active center was removed, and the target protein was hydrogenated and converted to pdbqt format using AutoDock software (13). The rotation key of the drug small molecules was set and saved in pdbqt format, the corresponding box parameters were set, and finally Vina was used for docking (14). Binding energy <0 indicated that the compound and protein could spontaneously bind and interact with each other. The lower the energy, the more stable the molecular conformation. Generally, a binding energy of ≤ 5.0 kcal/mol indicates a good binding effect. PyMol 2.3.2 software was used for visualization (15).

### Animals and CY-Induced Immunosuppression

Forty-eight Sprague-Dawley male rats were purchased from SPF (Beijing) Biotechnology Co., Ltd. (License No. SCXK[Jing]2019-0010). The average body weight (BW) of the rats was 200 ± 20 g. All animal procedures were conducted in accordance with the principles of proper laboratory animal care. The experiment was approved by the ethical review committee of Baotou Medical College (approval number: Baotou Medical Lun Audit Animal 2021 No. 017). The rats raised at a controlled at a controlled temperature of 20–25°C and relative humidity of 50 ± 10% with a 12/12 h light/dark cycle. The experiments were conducted after acclimatization for 1 week.

The experimental rats were divided into six groups, each group consisting of eight rats (Table 2). During the modeling period, except for the normal control rats, which received 200 μl of saline, rats in the other five groups were injected with a single intraperitoneal dose of cyclophosphamide (CY) at 100 mg/kg BW. Six hours after the CY treatment, the rats in the CY+H, CY+M, and CY+L groups were gavaged with koumiss at doses of 31.25, 20.83, and 10.42 ml/kg BW, respectively, corresponding to an adult human dose of 100, 200, and 300 ml of koumiss per day, respectively (adult weight was considered as 60 kg). The positive control group was administered Zhenqi Fuzheng capsule (immune booster/immune function enhancer) at a dose of 250 mg/kg BW (the dose setting was based on the conversion of human dose to animal dose).

**Table 2:** List of the six groups of rats.

**Group**	**Modeling period**		**Dosing period**	
	**Dose**	**Administration route**	**Dose**	**Administration route**
Normal	Physiological saline	Intraperitoneal injection	Distilled water	Oral
Model	Cyclophosphamide (100 mg/kg bw)	Intraperitoneal injection	Distilled water	Oral
Positive	Cyclophosphamide (100 mg/kg bw)	Intraperitoneal injection	Zhenqi fuzheng capsules (250 mg/kg bw)	Oral
CY+H	Cyclophosphamide (100 mg/kg bw)	Intraperitoneal injection	Koumiss (31.25 ml/kg bw)	Oral
CY+M	Cyclophosphamide (100 mg/kg bw)	Intraperitoneal injection	Koumiss (20.83 ml/kg bw)	Oral
CY+L	Cyclophosphamide (100 mg/kg bw)	Intraperitoneal injection	Koumiss (10.42 ml/kg bw)	Oral

### Organ Index Determination and Counting of Intestinal Peyer's Knots

Twenty-four hours after the last drug administration, the animals were weighed and sacrificed, following which the immune organs, including the thymus and the spleen, were immediately weighed. The thymus and spleen indices were calculated according to the following formula:


$$\begin{array}{l} \text{Index }\left(\text{mg}/\text{g}\right)=\left(\text{weight of thymus or spleen}\right)/\text{body weight}. \end{array}$$

The small intestine was removed from the rat and placed on a petri dish containing phosphate-buffered saline (PBS) solution to remove the fatty tissue from the surface of the intestinal mucosa. The number of Peyer's knots in the small intestine were visually observed and counted, and the average number of Peyer's knots in each group was recorded.

### Hematological Analyses

Blood was collected from all the animals *via* the abdominal aorta, and the following parameters were determined using an automated hematoanalyzer: (a) red blood cell count (RBC), (b) total white blood cell count (WBC), (c) monocyte count (MONOC), (d) platelet count (PC), (e) lymphocyte count (LYC), and (f) neutrophil count (NEUTC).

### Histological Analysis

The duodenum, jejunum, and ileum tissues preserved in 4% paraformaldehyde were removed and placed in an embedding box, and sections were obtained by alcohol dehydration, transparency, wax immersion, embedding and sectioning, followed by hematoxylin-eosin (HE) staining, air-drying, microscopic observation, and photography.

### Peripheral Blood Immunophenotyping Detection

Lymphocytes were separated using a lymphocyte separation medium and washed with PBS. The cells were diluted with PBS to a concentration of 1.0 × 10^6^ cells/ml. To label the cells, 10 μl of CD3^+^-FITC, CD4^+^-APC, and CD8^+^-PE monoclonal antibodies was added, and the solution was mixed well and placed for 15 min away from light. The cells were then washed and resuspended in PBS, and flow cytometry analysis was performed using a BD FACSCanto™ II (BD Biosciences) system.

### Statistical Analysis

All statistical data were expressed as the mean ± standard deviation if they conformed to normal distribution and the variance was uniform, and one-way ANOVA was used for comparison between multiple groups; if the variance was not uniform and the data did not conform to normal distribution, W-H rank sum test was used. All data were statistically analyzed with SPSS 19.0 software. Differences were considered significant at *P* < 0.05.

## Results

### Qualitative Analysis of Koumiss via LC-MS/MS

**Figure 2:**
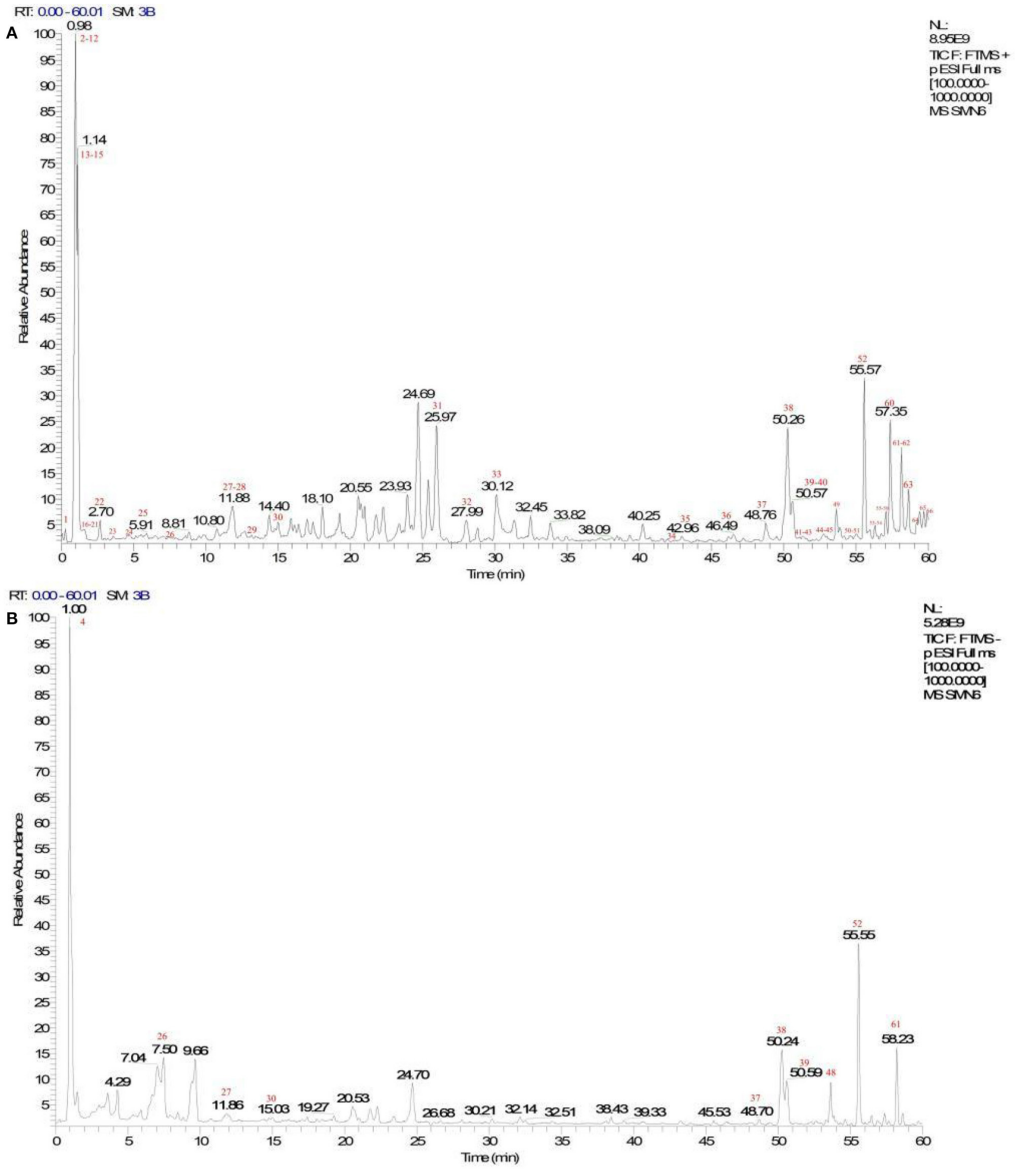
Total ion chromatograms of koumiss in the positive ionization mode (**A)** and negative ion mode **(B)**.

A total of 66 components were identified. The fragment ions were further identified using the Mass Frontier Fragmentation Library^TM^ for structural attribution. Ultimately, 21 compounds were identified using Compound Discoverer software and Mass Frontier Fragmentation Library^TM^ together. The total ion chromatograms of koumiss in positive ion mode and negative ion mode are presented in Figure 2. The retention times, molecular formulae, formulae, and DeltaMass of the 21 preliminarily identified compounds are shown in Table 3.

**Table 3:** Retention time, molecular formula, formula, and DeltaMass of the 66 identified compounds.

**No**.	**Name**	**RT (min)**	**Formula**	**Annot. DeltaMass (ppm)**	**Calc. MW**
1	Benzothiazole	0.435	C7 H5 N S	−0.91	135.01415
2	DL-Arginine^*^	0.984	C6 H14 N4 O2	−0.67	174.11156
3	Choline^*^	0.997	C5 H13 N O	3.15	103.10004
4	DL-Glutamine^*^	1.011	C5 H10 N2 O3	−0.11	146.06913
5	Creatine	1.035	C4 H9 N3 O2	0.25	131.06951
6	D-(+)-Proline^*^	1.049	C5 H9 N O2	1.57	115.06351
7	Threonine^*^	1.06	C4H9NO3	0.32045	119.05824
8	Acetylcholine	1.07	C7 H15 N O2	−0.64	145.11019
9	L(-)-Carnitine	1.121	C7 H15 N O3	−0.84	161.10506
10	Adenine	1.126	C5 H5 N5	−0.37	135.05445
11	Acetyl-L-carnitine	1.133	C9 H17 N O4	−0.47	203.11566
12	Valylproline^*^	1.138	C10 H18 N2 O3	−0.5	214.13163
13	L-Norleucine^*^	1.14	C6 H13 N O2	0.18	131.09465
14	Leucine^*^	1.14	C6H13NO2	0.17782	131.09462
15	Tyrosine^*^	1.18	C9H11NO3	−0.38058	181.07389
16	Tyramine	1.564	C8 H11 N O	0.66	137.08415
17	L-Isoleucine^*^	1.64	C6 H13 N O2	0.17	131.09465
18	Propionylcarnitine	2.033	C10 H19 N O4	−0.76	217.13124
19	6-Aminocaproic acid	2.275	C6 H13 N O2	−0.05	131.09462
20	Hypoxanthine	2.396	C5 H4 N4 O	−0.19	136.03848
21	Alanyltyrosine	2.407	C12 H16 N2 O4	−1.14	252.11072
22	L-Phenylalanine^*^	2.690	C9 H11 N O2	−0.35	165.07892
23	Leucylproline^*^	3.991	C11 H20 N2 O3	−0.29	228.14733
24	D-(+)-Tryptophan^*^	4.522	C11 H12 N2 O2	−0.64	204.08975
25	4-Acetamidobenzoic acid	5.902	C9 H9 N O3	−3.82	179.05756
26	Phenylacetylglycine	7.532	C10 H11 N O3	−2.07	193.07349
27	Valine^*^	11.852	C5 H11 N O2	1.72	117.07918
28	Tert-Butyl N-[1-(aminocarbonyl)-3-methylbutyl]carbamate	11.852	C11 H22 N2 O3	−1.39	230.16272
29	Hexanoylcarnitine	13.063	C13 H25 N O4	−0.81	259.17815
30	L-(-)-Methionine^*^	15.02	C5 H11 N O2 S	−0.76	149.05094
31	Prolylleucine	25.968	C11 H20 N2 O3	−1.44	228.14706
32	2,4-Dimethylbenzaldehyde	28.09	C9 H10 O	−0.66	134.07308
33	N-Butylbenzenesulfonamide	30.42	C10 H15 N O2 S	−1.41	213.08205
34	(+/–)12(14)-DiHOME	42.338	C18 H34 O4	−0.29	314.24562
35	Bis(4-ethylbenzylidene)sorbitol	42.948	C24 H30 O6	−1.66	414.20355
36	Progesterone^*^	46.428	C21 H30 O2	−1.7	314.22405
37	Octadecanamine	48.755	C18 H39 N	−1.31	269.3079
38	α-Linolenic acid^*^	50.26	C18 H30 O2	−1.16	278.22426
39	Citral^*^	50.578	C10 H16 O	−1.2	152.11993
40	16-Hydroxyhexadecanoic acid	50.59	C16 H32 O3	−0.65	272.23497
41	Monolaurin	51.149	C15 H30 O4	−1.21	274.21408
42	D-Sphingosine	51.342	C18 H37 N O2	−1.33	299.28203
43	11(Z),14(Z),17(Z)-Eicosatrienoic acid	51.347	C20 H34 O2	−1.71	306.25536
44	13(S)-HOTrE	52.77	C18 H30 O3	−1.42	294.21908
45	4-Dodecylbenzenesulfonic acid	52.773	C18 H30 O3 S	−0.54	326.19139
46	Myristyl sulfate	52.965	C14 H30 O4 S	−0.16	294.18643
47	Linoleic acid^*^	53.63	C18H32O2	−1.87746	280.24023
48	Pinolenic acid	53.707	C18 H30 O2	−1.22	278.22424
49	Dibutyl phthalate	53.894	C16 H22 O4	−2.07	278.15123
50	1,2-Dihydroxyheptadec-16-yn-4-yl acetate	54.509	C19 H34 O4	−1.95	326.24507
51	Lauric acid	54.649	C12 H24 O2	−4.21	200.17679 52
52	2,3-Dihydroxypropyl 12-methyltridecanoate	55.549	C17 H34 O4	−2.37	302.24499
53	Palmitoleic acid^*^	56.305	C16 H30 O2	−1.38	254.22423
54	Arachidonic acid	56.383	C20 H32 O2	−1.43	304.23979
55	Cis-12-Octadecenoic acid methyl ester	56.869	C19 H36 O2	−1.53	296.27108
56	Palmitoyl ethanolamide	57.026	C18 H37 N O2	−1.74	299.28191
57	Linolenic acid ethyl ester	57.042	C20 H34 O2	−1.91	306.2553
58	1-Linoleoyl glycerol	57.061	C21 H38 O4	−2.06	354.27628
59	2-Arachidonoyl glycerol	57.173	C23 H38 O4	−1.98	378.27626
60	α-Eleostearic acid	57.37	C18 H30 O2	−1.22	278.22424
61	Trans, trans-2,4-Heptadienal	58.096	C7 H10 O	0.93	110.07327
62	Hexadecanamide	58.346	C16 H33 N O	−1.75	255.25577
63	Docosapentaenoic acid	58.637	C22 H34 O2	−1.31	330.25545
64	Stearoyl ethanolamide	59.379	C20 H41 N O2	−1.84	327.31313
65	Palmitic acid^*^	59.687	C16 H32 O2	−1.24	256.23991
66	Ethyl palmitoleate	59.957	C18 H34 O2	−1.57	282.25544

* *Application of Mass Frontier Fragmentation Library^TM^, a small-molecule fragmentation mechanism library, for structural attribution of fragment ions*.

**Figure 3:**
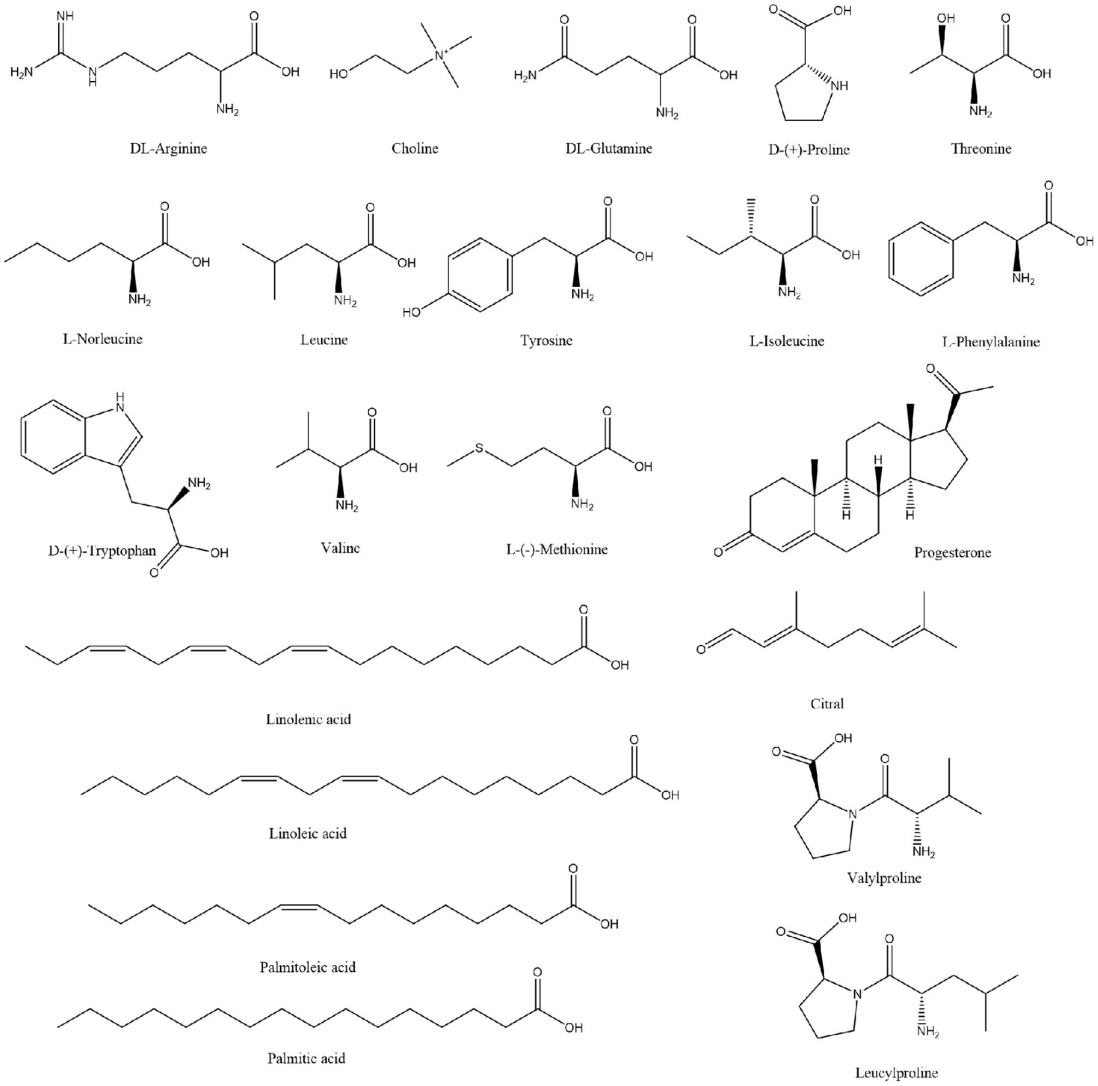
Chemical structure of the components tentatively identified in koumiss.

Extensive research has been conducted on the composition of koumiss, and the results of this experimental study revealed 66 main compounds, which is consistent with the findings of previous reports (16). Of these, 21 compounds were identified using Compound Discoverer software and Mass Frontier Fragmentation Library^TM^ together. Their structures are shown in Figure 3. Individual compounds in koumiss were characterized as follows: linolenic acid showed fragments at m/z 149, 123, and 135; peak 24 at m/z 204 was identified as tryptophan, as it showed fragments at m/z 188, 132, 118, and 159. This fragmentation pattern was consistent with that of the standard compounds, as well as patterns reported in previous studies. Based on data obtained from MS, MS/MS analysis, and literature, linoleic acid (peak 47) was detected in koumiss.

### Molecular Docking

**Figure 4:**
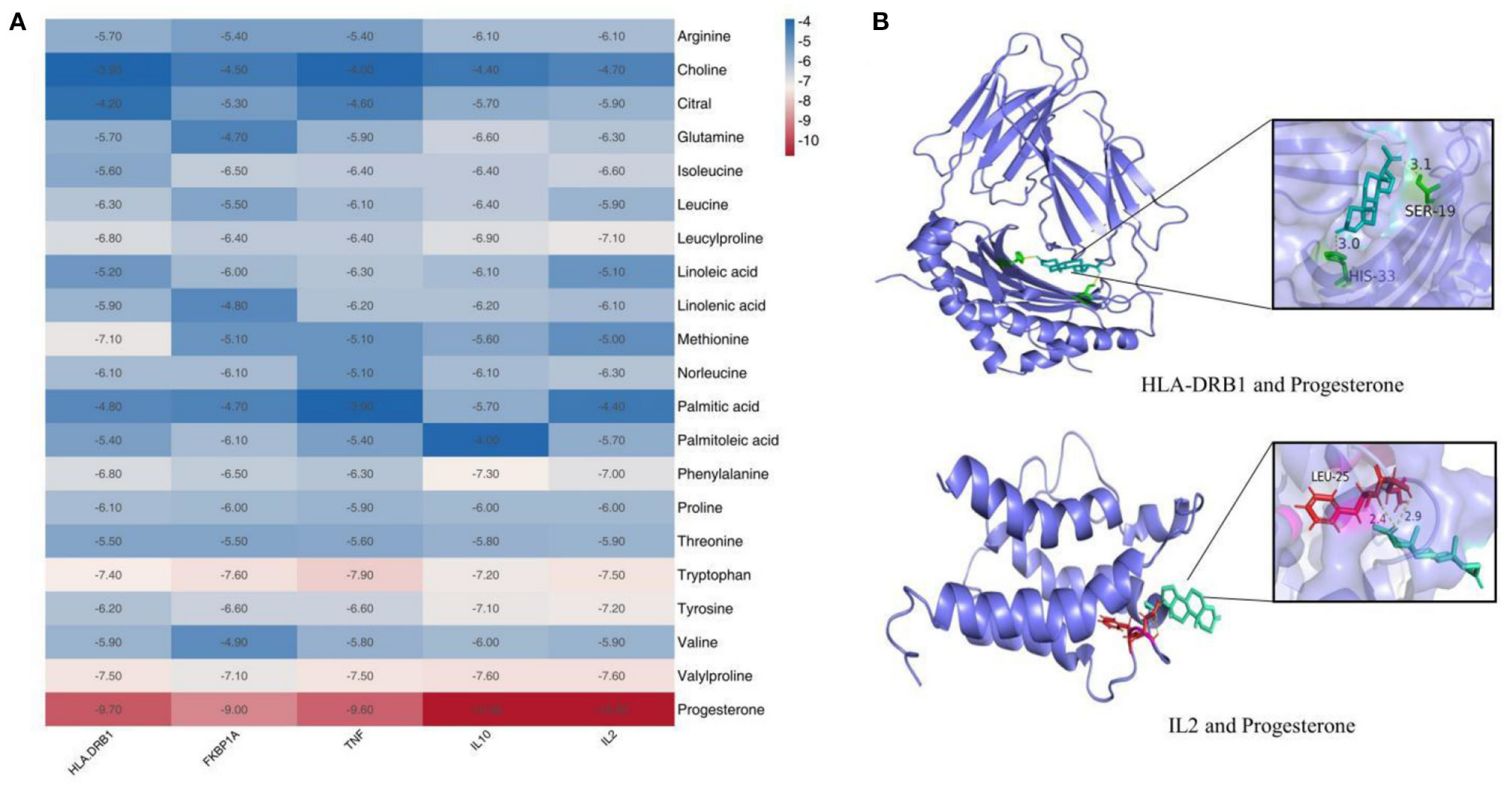
Molecular docking results of compounds in koumiss with immune-related targets. Binding energy thermogram of compounds identified in koumiss docked with immune-related target molecules **(A)**. Model diagram of compound-target molecule docking **(B)**.

Prior studies that have noted the importance of koumiss for immune modulation (8). Therefore, we evaluated the binding ability of the components of koumiss for immune-related targets. We selected five immunologically relevant targets from the Genecards database and 21 compounds identified using Compound Discoverer software and Mass Frontier Small Molecule Fragment Library^TM^ for molecular docking. The docking result is depicted as a heat map display in Figure 4A. The docking visualization results are shown in Figure 4B. The binding energy is related to the conformational fit of the binding site, hydrophobic interactions, hydrogen bonding linkage and van der Waals forces. The results showed that the binding energies of most of the compounds were < -5 kcal/mol, indicating that the compounds were well-bound to the proteins. Among them, the binding energies of IL2 and IL10 to progesterone were both −10.8 kcal/mol. The binding energies of D-(+)-Tryptophan and TNF were −7.9 kcal/mol.

### Effect of Koumiss on Organ Index and Peyer's Patches in CY-Treated Rats

Since the thymus and spleen are important immune organs and their indices can reflect the immune function of the organism, we further evaluated the organ indices of each group of rats. The results presented in Table 4 show that the spleen and the thymus indices were significantly lower in the model group than in the normal group (*P* < 0.01). The thymus index was markedly elevated by Zhenqi Fuzheng capsule treatment (*P* < 0.05). There were significant differences in spleen indices between koumiss-treated rats (10.42 and 20.83 mL/kg BW) and model group rats. This result is significant at the *P* < 0.05 level. No significant difference was observed in the thymus index between the koumiss-treated (20.83 and 31.25 mL/kg BW) and CY-treated groups.

**Table 4:** Spleen and thymus index of rats in each group (*n* = 8, *x* ± *s*).

**Group**	**Spleen index (mg/g)**	**Thymus index (mg/g)**
Normal	2.6019 ± 0.2105	1.8201 ± 0.1529
Model	1.9227 ± 0.2732^##^	1.1321 ± 0.2759^##^
Positive	2.3047 ± 0.4009	1.5111 ± 0.2822^*^
CY+L	2.1979 ± 0.1778^*^	1.5334 ± 0.1603^**^
CY+M	2.4611 ± 0.3816^**^	1.3718 ± 0.2215
CY+H	2.3134 ± 0.4624	1.4160 ± 0.2299

## *P < 0.01 vs. control group*;

* *P < 0.05*,

** *P < 0.01 vs. model group*.

**Figure 5:**
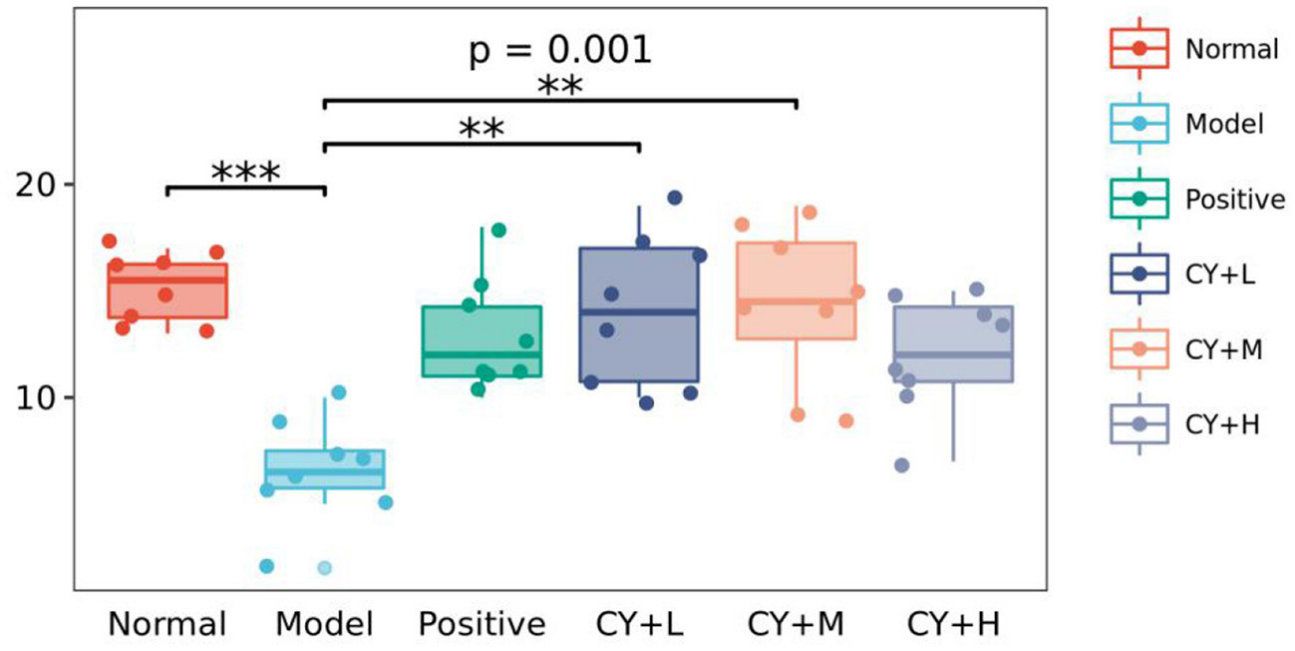
Effect of koumiss on intestinal Peyer's patches. The figure shows the *P*-values for the overall differences between groups obtained using the Kruskal–Wallis non-parametric test, and the markers for the significance levels of the differences obtained using the Dunn's test between groups (**P < 0.01; ***P < 0.001).

Figure 5 shows changes in the number of intestinal Peyer's patches in each group of rats. Compared to the normal group, the model group showed a significant reduction in the number of Peyer's patches (*P* < 0.05). The number of intestinal Peyer's patches in the koumiss-treated groups was significantly increased (CY+H group: *P* < 0.05, CY+M and CY+L groups: *P* < 0.01) compared to that in the model group. In summary, the results showed that atrophy of the thymus and spleen improved to some extent and the CY-induced immune organ damage in rats was alleviated by koumiss intervention. However, the damage did not revert completely to normal, considering that there were still some differences compared to normal rats.

### Effect of Koumiss on Hematological Parameters in CY-Treated Rats

Previous studies have demonstrated a decrease in the number of WBC and LYC in the serum of the body, indicating that the immune function of the body is affected (17). Therefore, we further tested the blood for various indicators. White blood cell count, RBC, PC, LYC, NEUTC, and MONOC were tested to determine the effect of koumiss on CY-induced immunosuppression (Table 5). White blood cell count, RBC, LYC, and NEUTC in the model group decreased significantly (*P* < 0.01, *P* < 0.05), while MONOC increased significantly (*P* < 0.05). Lymphocyte count, PC, and NEUTC in the positive (*P* < 0.01), CY+L (*P* < 0.05), CY+M (*P* < 0.05, *P* < 0.01), and the CY+H groups (*P* < 0.05, *P* < 0.01) were significantly higher than those in the model group. Additionally, LYC, PC, NEUTC, and MONOC were significantly higher in the CY+H group than in the model group (*P* < 0.05, *P* < 0.01). Thus, these results showed that koumiss treatment increased the number of WBC, PC, LYC, and MONOC, and alleviated immunosuppression.

**Table 5:** Comparison of blood index levels between the groups of rats (*n* = 8, *x̄* ± *s*).

**Group**	WBC (^*^10^**9**^/L)	RBC (^*^10^**9**^/L)	PC (^*^10^**9**^/L)	LYC (^*^10^**9**^/L)	NEUTC (^*^10^**9**^/L)	MONOC (^*^10^**9**^/L)
Normal	5.09 ± 1.30	9.22 ± 0.31	902.50 ± 47.91	4.11 ± 0.76	0.50 ± 0.10	0.12 ± 0.03
Model	3.97 ± 0.67^#^	8.12.09 ± 0.45^##^	1246.5 ± 184.49^##^	2.00 ± 1.03^##^	1.64 ± 1.00^##^	0.82 ± 1.09
Positive	8.22 ± 2.69^*^	7.91 ± 0.22	1325.63 ± 196.38	3.41 ± 2.39	3.40 ± 1.80^*^	1.32 ± 1.24
CY+L	6.96 ± 1.49^**^	7.64 ± 0.36^*^	1425.13 ± 77.02^*^	2.17 ± 1.52	3.53 ± 1.62^*^	1.43 ± 0.83
CY+M	7.85 ± 3.09^**^	7.71 ± 0.34	1509.50 ± 134.39^**^	1.44 ± 1.20	4.01 ± 2.11^*^	1.87 ± 1.05
CY+H	9.97 ± 3.16^**^	7.56 ± 0.21^**^	1669.88 ± 106.42^**^	2.00 ± 0.92	5.28 ± 2.72^**^	1.91 ± 1.28

# *P < 0.05*,

## *P < 0.01 vs. control group*;

* *P < 0.05*,

** *P < 0.01 vs. model group*.

### Effects of Koumiss on the Histopathology of CY-Treated Rats

**Figure 6:**
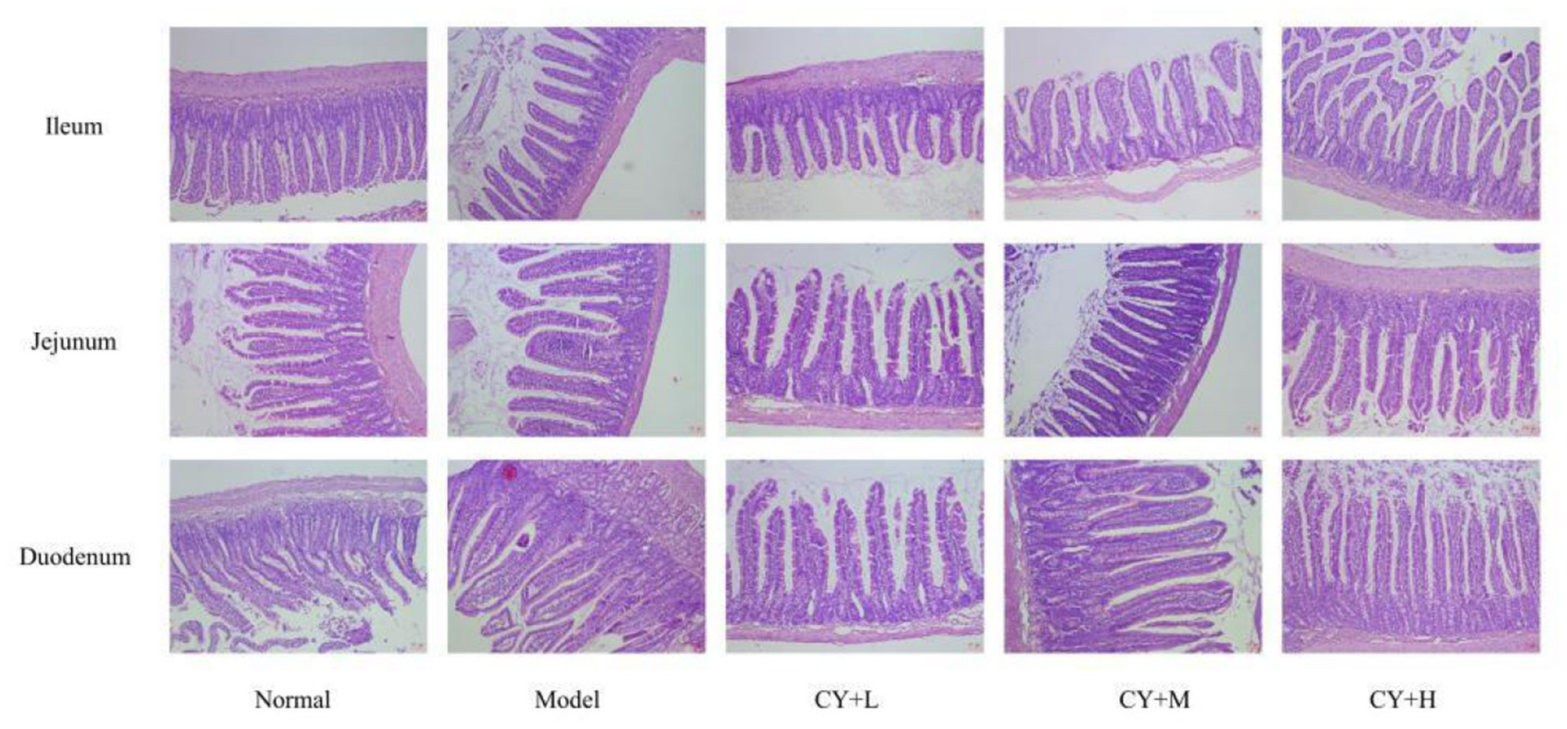
Effects of koumiss on the histomorphology of the ileum, jejunum, and duodenum in rats.

The main indicators of the health status of the small intestine include the depth of the small intestinal crypts, the height of the villi, and the ratio of the two, which were further investigated. The length of the villi in the jejunum and duodenum in each koumiss group was greater than that in the model group (Table 6), but the difference was not significant (*P* > 0.05). The crypt depths of the jejunum and duodenum were significantly lower than those of the model group (*P* < 0.05), and the differences in the crypt of the ileum were not significant (*P* > 0.05). The villus height to crypt depth (V/C) values were greater in the koumiss group than in the model group (*P* < 0.05), but the differences in V/C values in the jejunum were not significant (*P* > 0.05). These histopathological results showed that gavage with different doses of koumiss increased the villi length, decreased the crypt depth, and increased the V/C of each segment of the small intestine, thus effectively improving the mucosal structure of the small intestine. Figure 6 represents the effect of sour horse milk on the histomorphology of the ileum, jejunum, and duodenum of rats.

**Table 6:** The length of the villi in the jejunum and duodenum of each group (*n* = 3, *x̄* ± *s*).

**Group**	**Jejunum**			**Ileum**			**Duodenum**		
	**Length villous**	**Crypt depth**	**V/C**	**Length villous**	**Crypt depth**	**V/C**	**Length villous**	**Crypt depth**	**V/C**
	**V (μm)**	**C (μm)**		**V (μm)**	**C (μm)**		**V (μm)**	**C (μm)**	
Normal	355.01 ± 60.08	112.98 ± 8.75	3.16 ± 0.63	369.95 ± 28.15	122.43 ± 11.07	3.03 ± 0.14	316.53 ± 37.57	127.83 ± 15.86	2.48 ± 0.1
Model	326.61 ± 92.79	162.81 ± 86.25	2.18 ± 0.5	183.14 ± 29.63^##^	116.44 ± 12.31	1.57 ± 0.09^##^	404.03 ± 91.29	198.56 ± 24.09^##^	2.02 ± 0.23^##^
Positive	337.1 ± 98.16	158.92 ± 68.71	2.26 ± 0.48	236.89 ± 16.32	97.14 ± 12.18	2.46 ± 0.35^*^	385.22 ± 100.1	149.42 ± 20.45	2.56 ± 0.49
CY+L	366.92 ± 37.84	102.88 ± 19.24	3.66 ± 0.84	225.8 ± 10.46	92.63 ± 6.36^*^	2.45 ± 0.22^**^	381.53 ± 72.97	120.36 ± 28.67^*^	3.22 ± 0.59^*^
CY+M	334.67 ± 145.06	113.53 ± 36.66	2.92 ± 0.51	239.96 ± 24.59	92.28 ± 18.63	2.68 ± 0.68^*^	391.1 ± 138.21	147.91 ± 56.16	2.67 ± 0.1^*^
CY+H	367.9 ± 14.67	115.24 ± 18.21	3.24 ± 0.44	289.08 ± 33.57^*^	99.41 ± 22.44	2.95 ± 0.3^**^	410.37 ± 39.64	144.32 ± 52.08	3.01 ± 0.68

# *P < 0.05*,

## *P < 0.01 vs. control group*;

* *P < 0.05*,

** *P < 0.01 vs. model group*.

### Effects of Koumiss on Peripheral Blood Immunophenotyping in CY-Treated Rats

**Figure 7:**
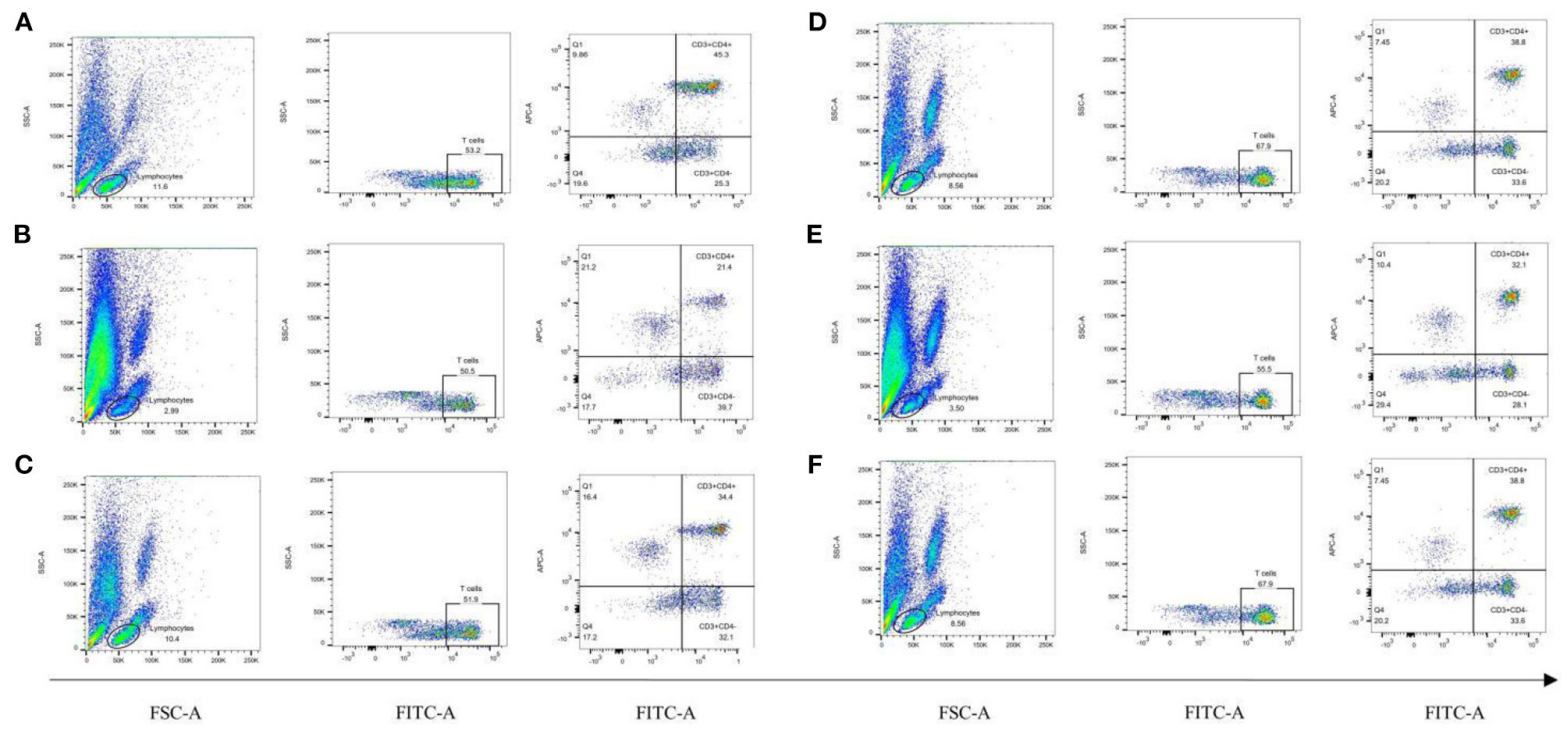
Proportions of CD3^+^, CD4^+^, and CD8^+^ cells in the peripheral blood lymphocytes of rats in each group. **(A)** Normal group, **(B)** Model group, **(C)** Positive group, **(D)** CY+L group, **(E)** CY+M group, **(F)** CY+H group.

To observe the distribution of T lymphocyte subpopulations in the peripheral blood of each group of rats, we further performed the assay using flow cytometry, as shown in Figure 7 and Table 7. The proportion of CD4^+^ cells and their ratio to CD8^+^ were significantly lower (*P* < 0.01) and the proportion of CD8^+^ cells was significantly higher (*P* < 0.01) in the model group, compared with the normal group (Table 7). CD4^+^ cells and its ratio to CD8^+^ cells in the positive group were significantly increased (*P* < 0.05) compared to the model group, and CD3^+^ was slightly increased but was not statistically significant (*P* > 0.05). Similarly, CD4^+^ and CD8^+^ levels in the koumiss-treated groups were significantly increased (*P* < 0.01) compared to those in the model group. CD3^+^ increased significantly in the CY+L group (*P* < 0.05), and the ratio of CD4^+^ to CD8^+^ cells increased significantly in the CY+M group (*P* < 0.05). These results showed that koumiss upregulated the expression of CD3^+^ and CD4^+^ cells in the peripheral blood lymphocytes and increased the CD4^+^/CD8^+^ ratio in model rats, which had a positive effect on the regulation of immunosuppression.

**Table 7:** Changes in blood T lymphocyte subsets of rats in each group (%).

**Group**	**CD3^**+**^**	**CD4^**+**^**	**CD8^**+**^**	**CD4^**+**^/CD8^**+**^**
Normal	36.3750 ± 5.9455	0.0122 ± 0.0010	0.0154 ± 0.0005	0.7925 ± 0.0713
Model	27.1625 ± 7.3856^#^	0.0098 ± 0.0015^##^	0.0167 ± 0.0007^##^	0.5848 ± 0.0958^##^
Positive	30.1875 ± 7.8103	0.0113 ± 0.0006^*^	0.0168 ± 0.0005	0.6736 ± 0.0396^*^
CY+L	37.9250 ± 4.6810^**^	0.0118 ± 0.0009^**^	0.0181 ± 0.0007^**^	0.6508 ± 0.0714
CY+M	29.2250 ± 6.1467	0.0123 ± 0.0011^**^	0.0179 ± 0.0005^**^	0.6878 ± 0.0669^*^
CY+H	33.1875 ± 7.6809	0.0118 ± 0.0008	0.0184 ± 0.0005^**^	0.6405 ± 0.0493

# *P < 0.05*,

## *P < 0.01 vs. control group*;

* *P < 0.05*,

** *P < 0.01 vs. model group*.

## Discussion

Mongolians have gained extensive clinical experience in the treatment of diseases with koumiss over a long period of time; however, the complete chemical characterization of koumiss and its role in modulation of immune function remain elusive. Therefore, the aim of this study was first to analyze the chemical properties of Koumiss using LC-MS method, and to explore the docking activity of the components of koumiss with immune-related targets using molecular docking technique, and finally to investigate the biological activity of koumiss in modulating intestinal immunity.

In this study, 21 compounds were identified using the UHPLC-Q Exactive high-resolution mass spectrometry platform in combination with fragment ion information from high-resolution secondary mass spectrometry, the Thermo Scientific mzCloud network database, the Mass Frontier Fragmentation Library^TM^, and relevant literature reports on the rapid analysis of chemical composition of koumiss, including amino acid and fatty acid components. These components have been shown to have immunomodulatory effects (18). IL-2, a T-cell growth factor, promotes the proliferation and activation of T cells and enhances the killing power of T cells. It has been reported that deficiencies of polyunsaturated fatty acids in mammals reduce lymphocyte proliferation and IL-2 production, and because the activity and function of lymphocytes are closely related to the metabolism of arachidonic acid, a deficiency of essential fatty acids in the diet reduces the immune function of lymphocytes (19). Thus, an appropriate amount of fatty acids in the diet can improve the immune function of the organism. In addition, studies on the role of lysine in the immune response have found that lysine can affect the immunity of the body. The addition of lysine to a 7% protein diet increases the level of immune antibodies against tetanus endotoxin in rats (20). Methionine has a major impact on the immune function of the animal organism. It has been reported that antibody responses to sheep red blood cells (SRBC) and delayed allergic responses to plant hemagglutination (PHA) are reduced in chicks with methionine deficient diets (21). The results of molecular docking similarly indicate that most of the compounds identified in koumiss have binding energies of < -5 kcal/mol to immune-related targets, and that these compounds bind well to proteins and can form more stable structures. The above results intuitively reflect that the components in koumiss may act on immune-related targets and thus exert immunomodulatory effects.

To further investigate the regulation mechanism of koumiss in immunity, we used a high-dose injection of CY (100 mg/kg) to establish an immunosuppressed rat model (22). CY is a drug that is commonly used in the preparation of immunosuppressed animal models; it causes leukopenia and immune organ weight loss through suppression of the bone marrow hematopoietic stem cells (23–25). The results showed that the growth rate of body, the thymus and spleen indices, number of Peyer's nodes, and the number of leukocytes in peripheral blood of CY-injected rats decreased significantly. In addition, there was a strong inhibitory effect on the functions of T and B lymphocytes and neutrophils, indicating that the immunosuppressed rat model was successfully established, which is consistent with the results of many related studies (26–28).

Immune organs, cells, and molecules together constitute the immune system of the human body, and immune dysfunction often leads to diseases (29). The growth and development of T lymphocytes are closely related to the thymus and spleen, and T lymphocytes can directly kill target cells; therefore, the thymus and spleen indices reflect the strength of the body's immune system (30). In addition, the Peyer's node is the immune induction and antigen uptake site of the intestinal mucosal immune system, which plays a local role in immunity (31, 32). It is rich in many immune cells, including thymus-derived cells and T and B cells; its size and number can reflect the local immune status of the intestinal mucosa. Among them, the lymphocyte-mediated immune response is not only related to the proliferative capacity of T and B lymphocytes, but also to the cytokines secreted by them. For example, TNF-α can bind to TNF R1 or TNF R2 of cell membrane, induce the differentiation of precursor cells of monocyte macrophages, and induce a variety of immunomodulatory mediators (33). Molecular docking revealed the good binding ability of TNF to most of the identified compounds, suggesting that koumiss may achieve immunomodulatory effects in various aspects of humoral and cellular immunity by binding TNF. Overall, the thymus and spleen indices and the number of Peyer's node were significantly reduced in the model group of CY-treated rats. The thymus and spleen indices and the number of Peyer's lymph nodes in rats treated with acid horse milk increased to varying degrees, tending to those of the normal group of rats (34).

Lymphocytes can be divided into two types:(1) T lymphocytes (CD3^+^), which are mainly involved in cellular immunity and can be further divided into helper T lymphocytes (Th) and cytotoxic T lymphocytes (Tc), and (2) B lymphocytes (CD19^+^), which are mainly involved in humoral immunity (35). Among them, Th cells are also known as CD4^+^ cells because they express CD4 on their surface, and are activated upon reaction with peptide antigens delivered by MHC II, which is expressed on the surface of antigen-presenting cells. Tc cells, also known as CD8^+^ cells, express CD8 on their surface. It is generally accepted that the CD4^+^/CD8^+^ ratio reflects the state of the body's cellular immune function, and a decrease in this ratio indicates that the body is in a state of immunosuppression (36). The levels of erythrocytes and leukocytes also reflect immunity, as erythrocytes can recognize antigens and promote the phagocytosis of macrophages; leukocytes are involved in the defense response of the body (37). The results of this study showed that koumiss increased the number of leukocytes, platelets, lymphocytes, and monocytes; upregulated the expression of CD3^+^ and CD4^+^ cells in peripheral blood; and increased the CD4^+^/CD8^+^ ratio in the model rats. In addition, molecular docking showed similar results. IL2 is a cytokine secreted by activated CD4^+^ and CD8^+^ T lymphocytes and is important for the proliferation of T and B lymphocytes, and most of the identified compounds showed good docking activity with IL2 (38). This demonstrates that the components of Koumiss may act on immune-related targets, thereby affecting lymphocytes to exhibit potent immune-promoting effects.

The small intestine is an important site for the digestion and absorption of major nutrients. Poorly developed or damaged small intestinal mucosa can adversely affect the normal growth, development, and immune function of the body (39). The length of the small intestinal villi is positively correlated with the number of small intestinal epithelial cells, and a change in the villi length directly affects the villi surface area (40). Additionally, regular oscillation of the intestinal villi also helps to exclude the colonization of harmful bacteria. The cells at the base of the crypt mature into epithelial cells, as they absorb nutrients during the upward migration to the villi. Upon reaching the villi, they supplement the shedding of the villi epithelium (41). A shallow crypt depth indicates an increase in the maturation rate of the intestinal epithelial cells and a reduced absorptive function. A high ratio of V/C indicates increased area of the small intestinal lining and enhanced digestive and absorptive capacity (42). The results of this study showed that, except for the villi length of the jejunum and duodenum, there was no significant difference between the groups. Gavage with different doses of koumiss increased the villi length, decreased the crypt depth, and increased the V/C of each segment of the small intestine, thus effectively improving the mucosal structure of the small intestine. These experimental results further suggest that when the body's immune function was low, koumiss improved the body's defense in a comprehensive manner from the perspective of non-specific and specific immunity, thus exhibiting a strong immune-promoting effect.

This study initially confirmed that koumiss can modulate intestinal immune function. However, if more comprehensive and reliable results are to be obtained, several gaps need to be addressed. First, the results of this study revealed that the intestinal villi of the model group rats were shortened and disorganized, which suggested that the intestinal mucosa of the model rats was damaged. This may lead to increased intestinal permeability and dysbiosis of the intestinal flora, resulting in the ectopic release of bacteria and endotoxins and further release of a series of inflammatory mediators, which may eventually lead to immune function impairment. It is therefore necessary to elucidate the mechanism of action of koumiss on intestinal flora in conjunction with microbiome analysis. Second, the available literature on the effect of koumiss on the prevention and treatment of diseases includes mostly clinical observations, lacking systematic, and rigorous experimental studies. Most patients need to use Mongolian or western medicine while consuming koumiss; it is thus impossible to judge the medicinal properties of koumiss; some literature has certain shortcomings in the evaluation of efficacy and data processing. Therefore, the physiological functions and the medical value of koumiss need to be better investigated with clinical data.

In summary, using the UHPLC-Q Exactive high-resolution mass spectrometry platform in combination with high-resolution secondary mass spectrometry and fragment ion information from Thermo Scientific mzCloud network database, 21 compounds were identified in koumiss. The compounds were analyzed through molecular docking with immune-related protein targets, and most of the compounds were found to have good binding activity to immune-related targets. In addition, koumiss can reduce the suppressive and destructive effects of CY on intestinal immune function by increasing the number of leukocytes, repairing the tissue structure of the spleen and thymus, and increasing the CD4^+^/CD8^+^ ratio via an immunosuppressive rat model. The preliminary findings of this experiment showed that koumiss had a significant positive effect on intestinal immune function in CY-induced immunocompromised rats, and the results of this study also provide basic data for further study of the mechanism of action and clinical application of koumiss.

## Data Availability Statement

The datasets presented in this study can be found in online repositories. The names of the repository/repositories and accession number(s) can be found in the article/supplementary material.

## Ethics Statement

The animal study was reviewed and approved by Bao Medical Lun Audit Animal 2021 No. (017).

## Author Contributions

ML conceived the research ideas. NZ and TX advised on data collection. QL, MJ, and XM designed the experiments in detail and provided valuable guidance on data analysis. ML and BS provided important insights and suggestions for this research. YZ aided in data consolidation. QL and CZ performed the majority of the data processing and wrote the manuscript. All authors significantly contributed to the manuscript and have read and approved the final manuscript.

## Funding

This work was supported by the National Natural Science Foundation of China (No. 81903925) and Science and Technology Program in Inner Mongolia (No. 201802097).

## Conflict of Interest

The authors declare that the research was conducted in the absence of any commercial or financial relationships that could be construed as a potential conflict of interest.

## Publisher's Note

All claims expressed in this article are solely those of the authors and do not necessarily represent those of their affiliated organizations, or those of the publisher, the editors and the reviewers. Any product that may be evaluated in this article, or claim that may be made by its manufacturer, is not guaranteed or endorsed by the publisher.
